# Quality improvement, implementation, and dissemination strategies to improve mental health care for children and adolescents: a systematic review

**DOI:** 10.1186/s13012-017-0626-4

**Published:** 2017-07-24

**Authors:** Valerie L. Forman-Hoffman, Jennifer Cook Middleton, Joni L. McKeeman, Leyla F. Stambaugh, Robert B. Christian, Bradley N. Gaynes, Heather Lynne Kane, Leila C. Kahwati, Kathleen N. Lohr, Meera Viswanathan

**Affiliations:** 1 RTI International, 3040 W Cornwallis Rd, Research Triangle Park, P.O. Box 12194, Research Triangle Park, NC 27709 USA; 2 Cecil G. Sheps Center for Health Services Research, The University of North Carolina at Chapel Hill, CB# 7590 725 Martin Luther King Jr. Blvd, Chapel Hill, NC 27599-7590 USA; 3 Department of Psychiatry, UNC School of Medicine, 235 Med Sch Wing C, 7160, Chapel Hill, NC 27599 USA; 4 The Carolina Institute for Developmental Disabilities, University of North Carolina Chapel Hill, Campus Box # 7255, Chapel Hill, NC 27599-7255 USA; 5 Department of Psychiatry, UNC School of Medicine, 304 MacNider Hall, CB #7160 333 S. Columbia Street, Chapel Hill, NC 27599 USA

**Keywords:** Adolescents, Children, Dissemination, Evidence-based medicine, Implementation, Mental health, Quality improvement, Systematic review

## Abstract

**Background:**

Some outcomes for children with mental health problems remain suboptimal because of poor access to care and the failure of systems and providers to adopt established quality improvement strategies and interventions with proven effectiveness. This review had three goals: (1) assess the effectiveness of quality improvement, implementation, and dissemination strategies intended to improve the mental health care of children and adolescents; (2) examine harms associated with these strategies; and (3) determine whether effectiveness or harms differ for subgroups based on system, organizational, practitioner, or patient characteristics.

**Methods:**

Sources included MEDLINE®, the Cochrane Library, PsycINFO, and CINAHL, from database inception through February 17, 2017. Additional sources included gray literature, additional studies from reference lists, and technical experts. Two reviewers selected relevant randomized controlled trials (RCTs) and observational studies, extracted data, and assessed risk of bias. Dual analysis, synthesis, and grading of the strength of evidence for each outcome followed for studies meeting inclusion criteria. We also used qualitative comparative analysis to examine relationships between combinations of strategy components and improvements in outcomes.

**Results:**

We identified 18 strategies described in 19 studies. Eleven strategies significantly improved at least one measure of intermediate outcomes, final health outcomes, or resource use. Moderate strength of evidence (from one RCT) supported using provider financial incentives such as pay for performance to improve the competence with which practitioners can implement evidence-based practices (EBPs). We found inconsistent evidence involving strategies with educational meetings, materials, and outreach; programs appeared to be successful in combination with reminders or providing practitioners with newly collected clinical information. We also found low strength of evidence for no benefit for initiatives that included only educational materials or meetings (or both), or only educational materials and outreach components. Evidence was insufficient to draw conclusions on harms and moderators of interventions.

**Conclusions:**

Several strategies can improve both intermediate and final health outcomes and resource use. This complex and heterogeneous body of evidence does not permit us to have a high degree of confidence about the efficacy of any one strategy because we generally found only a single study testing each strategy.

**Trial registration:**

PROSPERO, CRD42015024759.

## Background

Approximately one in five children and adolescents living in the USA has one or more mental, emotional, or behavioral health disorders according to the *Diagnostic and Statistical Manual of Mental Disorders, Fourth Edition* (DSM-IV) criteria in any given year [1]. These disorders contribute to problems with family, peers, and academic functioning. They may exacerbate coexisting conditions and reduce quality of life. They also increase the risk of involvement with the criminal justice system and other risk-taking behaviors and suicide [2].

Several key publications in the mid- to late-1990s suggested that usual care in children’s mental health had, at best, no [3] and sometimes harmful effects [4]. Since then, mental health interventions that improve outcomes for children and adolescents with mood disorders, anxiety disorders, disruptive behavior disorders, psychotic disorders, eating disorders, and substance use disorders have been tested and shown to yield varying degrees of benefit [5, 6].

Despite advances in the evidence base [5, 7], some outcomes for children with mental health problems remain suboptimal. Reasons include issues with access to care and the failure of systems and providers to adopt established quality improvement (QI) strategies and interventions with proven effectiveness (e.g., evidence-based practices (EBPs)). Studies using nationally representative data on US adolescents show that only approximately one in five children with mental health problems receives services and only one third of treatment episodes are considered minimally adequate (at least four visits with psychotropic medication or at least eight visits without psychotropic medication) [8–10]. The current health care system continues to provide fragmented care to children and adolescents in numerous uncoordinated systems, rendering inefficiently the delivery of needed services [11]. Moreover, clinicians—particularly primary care practitioners—may lack the time, knowledge, or training to identify and treat or refer patients with mental health problems [12].

Given the gap between observed and achievable processes and outcomes, one way to improve the mental health care of children and adolescents is to adopt QI practices; another is to develop strategies to implement or disseminate interventions with known effectiveness. Such strategies target changes in the organization and delivery of mental health services [13, 14]. They seek to improve the quality of care and patient outcomes by closing the gap between research evidence and practice [15–17].

Some investigators consider implementation and dissemination strategies as a particular subset of initiatives to improve the quality of care. However, the field of implementation and dissemination is so new that the conceptual framework and terminology in relationship to QI efforts have not been fully standardized yet [18]. We do not take a position on the taxonomy of these terms. More information about the definitions used in this review can be found in the full report on this topic [19].The ultimate goal of these strategies is to improve patient health and service utilization outcomes for children and adolescents with mental health problems through system interventions, not clinical ones. Intermediate outcomes in this context include changes to health care systems, organizations, and practitioners that provide mental health care. Targeting multiple, interrelated, nested levels—such as the macro environment (e.g., states), organization or system (e.g., specialty mental health clinics), program (e.g., selected interventions), practitioner (e.g., psychologists), and patient (e.g., children or adolescents and their families)—typically increases the effectiveness and sustainability of a particular strategy [20, 21]. These outcomes represent implementation, dissemination, or QI outcomes and are distinct from but can influence patient-level outcomes [20], For instance, changes in intermediate outcomes such as practitioners’ attitudes [22] or organizational climate [23] may influence the successful adoption of and fidelity to EBPs. These practices in turn influence patient health outcomes, such as behavior or quality of life.

We developed the topic, key questions (KQs), outcome list, and analytic framework for this systematic review through a comprehensive, evidence-based, and public process. We refined review criteria used by two recent reviews [24, 25] and built on extant literature [20–23, 26–28] on this topic to focus the review on a narrower set of inclusion and exclusion criteria. We aimed to decrease the heterogeneity of findings, add more recent studies, and seek studies that may have examined differential efficacy by patient, provider, or system-level characteristics. A panel of key informants gave input on the proposed scope and methodology. Public comment was solicited in response to online posting on AHRQ’s Effective Health Care Website. We revised the KQs, outcomes, and analytic framework in response to all gathered information.

We drafted a protocol for the systematic review and recruited a panel of technical experts to provide high-level content and methodological consultation throughout the review. The final protocol was posted on the Effective Health Care website at http://effectivehealthcare.ahrq.gov/search-for-guides-reviews-and-reports/?pageaction=displayproduct&productid=2030 on December 30, 2014, and registered on PROSPERO (Registration number: CRD42015024759). Following release of a draft of the systematic review and peer review, we amended our protocol to include additional review and analysis methods suitable for complex interventions (described below in the “Methods” section).

Our key questions included the following:

- KQ 1: What is the effectiveness of quality improvement (QI), implementation, and dissemination strategies employed in outpatient settings by health care practitioners, organizations, or systems that care for children and adolescents with mental health problems to improve:
  - Intermediate patient, provider, or system outcomes
  - Patient health and service utilization outcomes?

- KQ 2: What are the harms of these mental health strategies?
- KQ 3: Do characteristics of the child or adolescent or contextual factors (e.g., characteristics of patients, practitioners, organizations, or systems; intervention characteristics; setting; or process) modify the effectiveness or harms of strategies to improve mental health care and, if so, how?

**Fig. 1:**
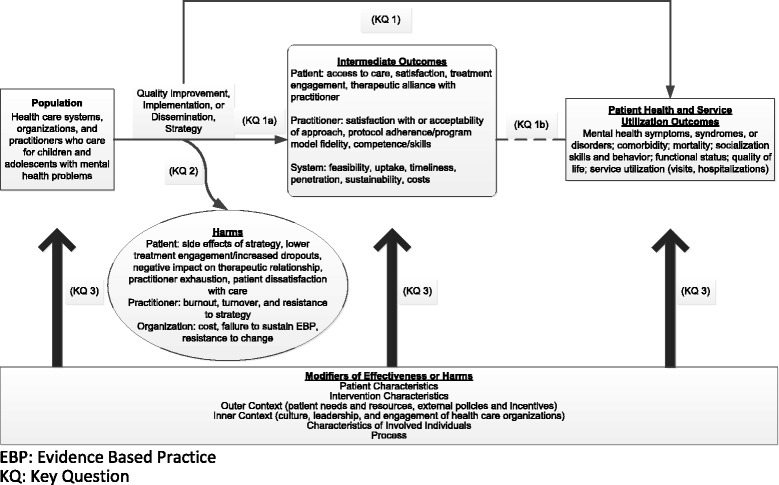
Analytic framework for strategies to improve mental health care in children and adolescents

Figure 1 depicts our analytic framework. Note that all KQ focus on the effectiveness, harms, and moderators of outcomes (effectiveness or harms) of strategies to implement, disseminate, or improve quality of mental healthcare for children and adolescents. The benefits and harms of strategies accrue at multiple levels: systems or organizations, practitioners, and patients. Clinical interventions that focused solely on improving health outcomes were not eligible.

KQ 3 was intended to evaluate the effect of moderators on any outcomes reported in KQ 1 (either intermediate or ultimate patient health or service utilization benefits to systems or organizations, practitioners, or patients) or KQ 2 (harms to systems or organizations, practitioners, or patients). Because the effect of the moderator may vary based on the nature of the outcome, our planned analyses did not combine categories of outcomes.

## Methods

We followed procedures specified in the *Methods Guide for Effectiveness and Comparative Effectiveness Reviews* from AHRQ (available at https://www.effectivehealthcare.ahrq.gov/search-for-guides-reviews-and-reports/?pageaction=displayproduct&productid=318). The review uses the PRISMA (Preferred Reporting Items for Systematic Reviews and Meta-Analyses) checklist to facilitate the preparation and reporting of the systematic review [29].

### Searches

We searched MEDLINE® for eligible interventions using a combination of Medical Subject Headings (MeSH), abstract keywords, and words or phrases found in the title of the paper, limiting the search to human-only studies, from database inception through February 17, 2017. We also searched the Cochrane Library, PsycINFO, and CINAHL (Cumulative Index to Nursing and Allied Health Literature) using analogous search terms. The full report [19] provides a full electronic search strategy for each database searched, but it includes a synthesis of findings only from studies meeting review criteria that we had identified via searches conducted through January 14, 2016.

In addition, we searched the gray literature for eligible studies. Sources of gray literature include ClinicalTrials.gov, the World Health Organization’s International Clinical Trials Registry Platform, the National Institutes of Health Research Portfolio Online Reporting Tools, the Database of Promoting Health Effectiveness Reviews, and CMS.gov. To avoid retrieval bias, we manually searched the reference lists of landmark studies and background articles on this topic to look for any relevant citations that our electronic searches might have missed.

### Study inclusion and exclusion criteria

We specified our inclusion and exclusion criteria based on the patient populations, interventions, comparators, outcomes, timing of outcomes assessment, and setting (PICOTS) we identified as relevant during our scope development through the topic refinement process (Table 1). We included QI, implementation, and dissemination strategies that targeted systems, organizations, or practitioners that deliver mental health care to children and adolescents who were already experiencing mental health symptoms. As a result, we did not include universal interventions aimed at prevention. We also did not include strategies such as the implementation of educational interventions for reading disorders. We required that implementation strategies focus on EBP interventions. For defining EBPs, we relied on the minimum requirements set forth in the National Registry of Evidence-based Programs and Practices (NREPP) from the Substance Abuse and Mental Health Services Administration (www.nrepp.samhsa.gov). These criteria specify that the intervention needs to have produced one or more positive behavioral outcomes in at least one study using an experimental or quasi-experimental design with results published in a peer-reviewed journal or similar publication. In addition, implementation materials, training and support resources, and quality assurance procedures for these interventions need to be ready for use.

**Table 1:** Inclusion/exclusion criteria for strategies to improve mental health services for children and adolescents

Category	Inclusion	Exclusion
Population	Health care systems, organizations, and practitioners that care for children and adolescents or mixed (child and adult) populations with mental health problems	• Health care systems, organizations, and practitioners that care only for adults 18 years of age or older • Health care systems, organizations, and practitioners that care for children and adolescents with only developmental disorders
Interventions (Strategies)	• Quality improvement strategies (e.g., strategies targeting systems and practitioners of mental health care to children and adolescents with the goal of improved quality of care)	Interventions targeting only patients, only drug interventions (although strategies to implement or disseminate drug interventions would qualify), and interventions not otherwise described in inclusion criteria
	• Implementation strategies (e.g., strategies to integrate evidence-based practice (EBP) interventions that meet National Registry of Evidence-based Programs and Practices (NREPP) inclusion criteria with the goal of changing practice patterns)	
	• Dissemination strategies (e.g., strategies to enhance the adoption and implementation of evidence-based interventions that meet NREPP inclusion criteria)	
Comparator	Any control strategy, including usual care or different variants of the same intervention	None
Outcomes	**Intermediate outcomes** (at least one intermediate outcome is required for KQs 1, 3)	All outcomes not otherwise specified
	*Patient*	
	• Access to care • Satisfaction • Treatment engagement • Therapeutic alliance with practitioner	
	*Practitioner*	
	• Satisfaction with or acceptability of approach • Protocol adherence/program model fidelity • Competence or skills	
	*System or organization*	
	• Feasibility • Uptake • Timeliness • Penetration • Sustainability • Resources (including costs)	
	**Patient health and service utilization outcomes** (at least one of these outcomes is required for KQs 1 and 3 unless the strategy uses an intervention that is an EBP)	
	*Required:*	
	• Change in mental health status, including symptom change, response, remission, relapse, and recurrence	
	• Coexisting physical health conditions, substance use problems, developmental disorders, other mental health problems	
	*Not Required:*	
	• Mortality • Socialization skills and behavior • Functional status • Quality of life • Service utilization (e.g., visits, hospitalizations)	
	**Harms of strategy**	
	*Patient*	
	• Lower treatment engagement or more dropouts • Negative impact on therapeutic relationship • Side effects of EBP incorporated into strategy (e.g., adverse events, suicidality)	
	*Practitioner*	
	• Burnout or exhaustion • Turnover • Resistance to the intervention	
	*System or organization*	
	• Cost • Failure to sustain the EBP • Resistance to change	
Timing of outcome measurement	All	None
Settings	Outpatient settings serving children and adolescents with mental health problems (primary care, specialty care, emergency rooms, community mental health centers, integrated care settings, federally qualified health centers, schools, homes)	Inpatient or residential treatment settings, drug treatment programs, jails or prisons
Geographic setting	Countries with a very high Human Development Index (HDI) [76]	Countries with high, medium, low, or very low HDI
Publication language	English	All other languages
Study design	**KQs 1, 3 (benefits)**	Case series Case reports Nonsystematic reviews Cross-sectional studies Before and after studies without time-series data Other designs without a control or comparison group
	• RCTs • CCTs • Systematic review and meta-analyses • Cohort studies • Interrupted time series • Case-control studies	
	**KQs 2, 3 (harms):**	
	• RCTs • CCTs • Systematic review and meta-analyses • Cohort studies • Interrupted time series • Case-control studies	
Publication type	Any publication reporting primary data	Publications not reporting primary data

*CCT* controlled clinical trial, *EBP* evidence-based practice, *D* dissemination *HDI* Human Development Index, *I* implementation, *KQ* key question, *NREPP* National Registry of Evidence-based Programs and Practices, *QI* quality improvement, *RCT* randomized controlled trial

We use the term “strategy” to mean the approach used to target health care systems, practitioners, or both to improve the quality of care for children and adolescents with mental health problems. We use the term “intervention” to denote a specific EBP used as part of a strategy.

Because strategies tended to be complex in nature and the number and types of components that varied between the treatment arm and comparison group arm differed by study, we also recorded components of each strategy. We relied on the Cochrane Review Group’s Effective Practice and Organization of Care (EPOC) Group taxonomy, which categorizes strategies by whether they include one or more professional, financial, organizational, and regulatory components [30].

Because many comparison group strategies also had several components, we noted and compared the components in each study arm. This allowed us to describe fully the numerous components that were being combined and tested in each strategy; it also enabled us to determine whether the study arms differed by a single or multiple components.

We required each included study to report at least one intermediate outcome in a minimum of one of three major categories: (1) practitioner intermediate outcomes (satisfaction, adherence, fidelity, competence), (2) system intermediate outcomes (feasibility, uptake, timeliness, penetration, sustainability, costs), and (3) patient intermediate outcomes (access to care, satisfaction, engagement, therapeutic alliance). Harms of interest included those at the patient, provider, and/or system that are potentially associated with the strategies themselves.

As noted earlier, the choice of outcomes in the review was based on existing evidence and theory [31], feedback from key informants, and input from the public. This approach helped ensure that each included study demonstrated impact based on its stated goals of improving quality or implementing or disseminating evidence-based interventions. We also required each study to report at least one patient health or service utilization outcome (change in mental health status, comorbid conditions, mortality, socialization skills and behavior, functional status, quality of life; service utilization) if the strategy was not implementing or disseminating an EBP (i.e., an intervention with proven effectiveness).

For all KQs, we excluded study designs without comparison groups to ensure that our pool of included studies provided strong evidence on the causal link between the strategy and outcomes. We excluded studies in which the strategy (system, organizational, practitioner targets) and the intervention being tested both differed between groups, because the effectiveness of the QI, implementation, or dissemination strategy could not be isolated from the baseline intervention effects.

Our exclusion of non-English-language studies is based on limitations of time and resources. However, we examined English language abstracts of non-English-language studies to assess the potential size of the literature that we might otherwise miss through this approach.

### Potential effect modifiers and reasons for heterogeneity

To begin to understand salient contextual factors and guide our analyses of effect modifiers, we applied the Consolidated Framework for Implementation Research (CFIR) [32] to research on effective implementation of mental health strategies for children and adolescents [33]. We searched for evidence on the modifying effects of each of the five domains of CFIR, intervention characteristics; outer setting (e.g., patient needs and resources, external policies); inner setting (e.g., culture, leadership, and engagement of health care organizations); knowledge, attitudes, and behaviors of involved individuals; and process characteristics (e.g., fidelity, use of champions, supervision or oversight). To this, we added a sixth category, patient characteristics.

### Study quality assessment

To assess the risk of bias (internal validity) of studies, two independent reviewers used predefined, design-specific criteria based on guidance in the *Methods Guide* [34]. We resolved conflicts by consensus or by consulting a third member of the team. For randomized controlled trials (RCTs), we relied on the risk of bias tool developed by the Cochrane Collaboration [35]. We assessed the risk of bias of observational studies using questions from an item bank developed by RTI International [36] and A Cochrane Risk Of Bias Assessment Tool for Non-Randomized Studies of Interventions (ACROBAT-NRSI) [37]. Minimum eligibility criteria for systematic reviews included an explicit description of search strategy used and determination that the search strategy was adequate, application of predefined eligibility criteria, risk of bias assessment for all included studies, and synthesis of the results presented.

In general terms, a study with no identifiable flaws has a low risk of bias. A study with medium risk of bias is susceptible to some bias but probably not sufficient to invalidate its results. A study with high risk of bias has significant methodological flaws (stemming from, for example, serious errors in design or conduct) that may invalidate its results. We considered the risk of bias for each relevant outcome of a study. When studies did not report sufficient detail to assess the validity of the design or study conduct, we judged the risk of bias to be unclear.

### Data extraction strategy

Trained reviewers abstracted important information from included studies into evidence tables, which were uploaded to AHRQ’s Systematic Review Data Repository. A second senior member of the team reviewed all data abstractions for completeness and accuracy. Reviewers resolved conflicts by discussion and consensus or by consulting a third member of the review team.

### Data synthesis and presentation

To determine whether quantitative analyses were appropriate, we assessed the clinical and methodological heterogeneity of the studies under consideration following established guidance [38]. For all outcomes, we present relative risks or mean differences, with confidence intervals (CIs), whenever calculable.

We employed several other methods to provide additional information about the nature of the strategies tested and what components of the strategies had the most impact on outcomes. First, we performed additional search approaches of related publications (known as “cluster searching”) to identify sibling (multiple publications on the same study) or kinship studies (publications from a common antecedent study or common theoretical foundation) [39]. We hoped to uncover contextual information to explain failure or success of strategies. We also contacted study authors to obtain information about critical components for strategies of included studies as part of a parallel project to understand better the uses and limitations of trial registries for data on outcomes. This effort provided additional information on the important components of the strategies tested in included studies. Finally, we used qualitative comparative analysis (QCA) to examine condition sets between combinations of strategy components to identify those that were most associated with improvements in outcomes [40, 41]. QCA is a data analytic technique that bridges quantitative and qualitative analyses by examining intervention components to permit analysts to infer which combinations relate to desirable and undesirable outcomes.

We graded the strength of a body of evidence based on the updated guidance in the *Methods Guide* [42, 43]. The AHRQ Evidence-based Practice Center (EPC) approach incorporates five key domains: study limitations, consistency, directness, precision of the evidence, and reporting bias. It also considers other optional domains that may be relevant for some scenarios, such as a dose-response association, plausible confounding that would decrease the observed effect, and strength of association (magnitude of effect). These domains are particularly relevant for observational studies.

Two reviewers assessed each domain for each key outcome and resolved any differences by consensus discussion*.* Senior members of the review team graded the strength of evidence.

Grades reflect the confidence that the reviewers have that various estimates of effect are close to true effects with respect to the KQs in a systematic review. A high grade indicates that we are very confident that the estimate of effect lies close to the true effect for this outcome. A moderate grade indicates moderate confidence that estimate of effect lies close to the true effect for this outcome and the body of evidence has some deficiencies. A low grade indicates limited confidence that the estimate of effect lies close to the true effect for this outcome. The body of evidence has major or numerous deficiencies. A grade of “insufficient” applies when we have no evidence, we are unable to estimate an effect, or we have no confidence in the estimate of effect for this outcome [42].

Risk of bias assessments for individual studies feed into the rating for the first strength of evidence domain, study limitations. Specifically, we rated bodies of evidence comprising trials with a high risk of bias as having high study limitations. Medium or unclear risk of bias studies resulted in medium study limitations. Low risk of bias studies resulted in low study limitations. In keeping with GRADE and strength of evidence guidance, we rated observational studies as having high study limitations [43, 44].

As described above, study design and study limitations together set the baseline strength of evidence grade. Other domains (inconsistency, imprecision, indirectness, reporting bias) then could either reduce or increase the grade. A body of evidence with high study limitations, with no other reasons to increase confidence (dose-response, large magnitude of effect, plausible confounding) or decrease it (inconsistency, imprecision, indirectness, reporting bias), would generally have a low strength of evidence grade. A body of evidence with low study limitations, with no reasons to decrease confidence (inconsistency, imprecision, indirectness, reporting bias), would generally have a high strength of evidence grade.

For each source of uncertainty, we consistently used the following rubric to evaluate its effect on the overall strength of evidence across outcomes. Specifically, for indirectness, we rated intermediate outcomes as direct, rather than indirect, evidence. Typically, strength of evidence grading systems will downgrade all evidence from intermediate outcomes as a matter of course. For this review, such an approach would have penalized strategies from studies whose intent was to examine implementation outcomes.

For this systematic review, these outcomes can be interpreted as direct measures of process change. Regarding inconsistency, we rated it as unknown for bodies of evidence with single studies; the rating of unknown consistency did not lower the overall grade. We relied on established guidance to judge imprecision [45]. Regarding imprecision, we specified the reasons for our judgment (small sample size or event rate, particularly when considering the optimum information size for the specific outcome, CIs crossing the line of no difference, or very wide CIs) [44]. We downgraded the overall strength of evidence by two levels when we found multiple reasons for imprecision. We upgraded the evidence by one level for factors such as large magnitude of effect.

## Results

We summarize results by KQ below for searches through February 17, 2017. The full report [19] provides detailed descriptions of included studies, key points, detailed synthesis, summary tables, and expanded strength of evidence tables for studies via original searches conducted through January 14, 2016.

### Review statistics

**Fig. 2:**
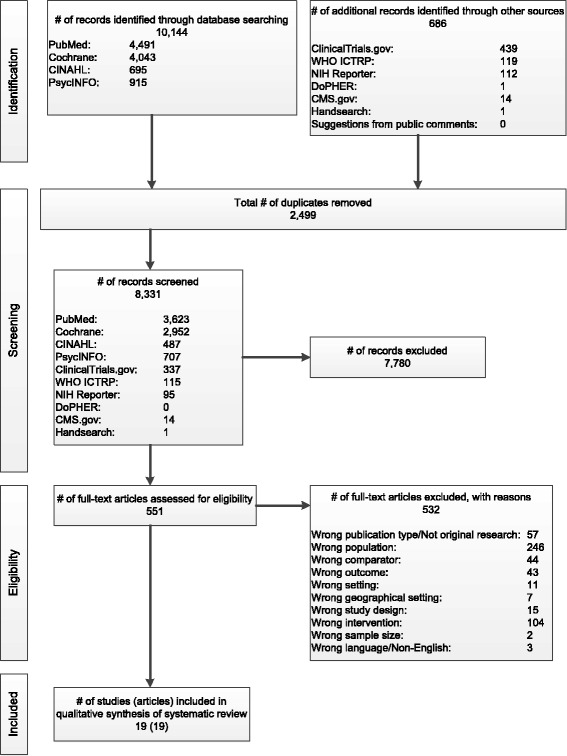
Results of literature searches for quality improvement, implementation, and dissemination strategies to improve mental health care for children and adolescents

Figure 2 presents our literature search results. We found 19 eligible articles representing 19 studies [13, 14, 46–62] (one article reported on two different studies [55] and another two articles [51, 60] reported outcomes for the same trial). These studies represent 18 strategies. We did not find any relevant non-English studies with English abstracts.

This evidence base for KQ 1 consisted of 19 studies [13, 14, 46–62]. Of these, one addressed KQ 2 (harms) and four addressed KQ 3 (moderators of effectiveness). No study addressed the moderators of harms. The evidence base included RCTs (some of which were cluster RCTs) [13, 14, 46–48, 50, 51, 53, 55–62], (nonrandomized) controlled clinical trials (CCTs) [52, 54], interrupted time series [49], and cohort designs [55]. Full evidence tables for articles identified via searches through January 14, 2016, are available at http://srdr.ahrq.gov/projects/530.

### Study quality assessment

Table 2 describes interventions and summarizes the evidence for included studies. Most strategies were complex and included multiple (two to seven) different components (as defined by the EPOC taxonomy). We graded the strength of more than half of the combined 49 outcomes assessed across all included studies as insufficient or low for no benefit.

**Table 2:** Strategies to improve mental health of children and adolescents: summary table

Strategy, study designs, *N*	Target condition and ages of youth	Comparisons	Component of the strategy	Major findings	Strength of evidence from results	Reasons for strength of evidence
Training therapists to implement an EBP Beidas et al. [50] Cluster RCT, 115 therapists	Anxiety Ages 8–17 years	Augmented active learning vs. routine professional training workshop	Educational meetings or materials	No differences between arms for practitioner satisfaction with approach, protocol adherence, or practitioner skill	Low for no benefit for practitioner satisfaction, adherence, and skill	Low risk of bias, small sample size, imprecise results
		Computerized routine training vs. routine professional training workshop	Educational meetings or materials	No differences between arms for practitioner protocol adherence or program model fidelity, or skill; computerized training group practitioners less satisfied than routine training group practitioners	Low for no benefit for practitioner satisfaction, adherence, and skill	Low risk of bias, small sample size, imprecise results
Feedback of patient symptoms to practitioners Bickman et al. [13] Cluster RCT, *N* of clinicians unclear, 340 youth, 144 clinicians, 383 caregivers	General mental health problem (children who receive home-based mental health treatment) Mean age = 15 years	Weekly and cumulative 90-day feedback vs. cumulative 90-day feedback only on patient symptoms and functioning to practitioners	Audit and feedback	Two thirds of practitioners did not view Web module	Insufficient for practitioner adherence	High study limitations, unknown precision for adherence
				Membership in the weekly feedback group increased the rate of decline in functional severity scale by 0.01 (range: 1 to 5, higher scores indicate greater severity)	Low for benefit for functional severity	High study limitations, precise results for symptoms
Feedback of patient treatment progress (symptoms and functioning) and process (e.g., therapeutic alliance) to practitioners Bickman et al. [61] Randomized block RCT, *N* of clinicians unclear, 257 youth, 2 clinics (one urban, one rural) at a single agency 21 clinicians, 255 caregivers	General mental health problem (children who receive mental health treatment from a community mental health clinic) New patients aged 11–18	Session-by-session feedback vs. cumulative 6-month feedback to clinicians	Audit and feedback	No significant differences in percentage of sessions held or percentage of clinicians, youth, or caregivers who completed the questionnaire required at each visit	Insufficient for patient engagement, for practitioner adherence/program model fidelity, and system uptake	High study limitations, unknown precision for each intermediate outcome.
				No patient-reported, caregiver-reported, or clinician-reported differences in symptoms or functioning of youth associated with intervention group in either clinic except feedback effects only seen in clinician ratings from one clinic (beta feedback*slope = −0.01, *p* = 0.045)	Low for no benefit for symptom severity	High study limitations, precise results for symptoms
Computer decision support for guidelines Carroll et al. [46] Cluster RCT, 84 patients	General mental health problem (children who receive home-based mental health treatment) Mean age = 15 years	Computer decision support plus electronic health record (EHR) that included diagnosis and treatment guidelines vs. computer decision support plus EHR only	Educational meetings or materials Patient-reported data Reminders Quality monitoring	Practitioner adherence improved through uptake of guidelines for diagnostic assessment (aOR, 8.0; 95% CI, 1.6 to 40.6); more reporting of 3 of 4 symptom domains at diagnosis	Low for benefit for practitioner adherence and program model fidelity	Medium study limitations, imprecise results with small number of events, large magnitude of effect
				No statistically significant differences on practitioner adherence through reassessment of symptoms at 3 months, adjustment of medications, and mental health referral	Insufficient for practitioner adherence (reassessment of symptoms) at 3 months, adjustment of medications, and referral	Medium study limitations, imprecise results (CIs cross the line of no difference)
				Visit to a mental health specialist calculated OR 2.195; 95% CI, 0.909 to 5.303; *p* = 0.081; reported *p* value in study = 0.054	Insufficient for service utilization	Medium study limitations, imprecise results (CIs cross the line of no difference)
Internet portal to provide access to practice guidelines Epstein et al. [56] Cluster RCT, 746 patients	Attention deficit hyperactivity disorder (ADHD) Ages 6 to 12 years	Internet portal providing practitioner access to practice guidelines vs. wait-list control	Educational meetings or materials Patient-reported data Audit and feedback Reminders Quality monitoring	Strategy appeared to improve 4 of 5 examined outcomes that measured practitioner protocol adherence and program model fidelity outcomes (mean change in proportion of patients who received targeted, evidence-based ADHD care outcomes between groups ranged from 16.6 to −50), but estimates were very imprecise, with large CIs	Low for benefit for practitioner protocol adherence and program model fidelity	Medium study limitations, imprecise (wide CIs)
Collaborative consultation treatment service to implement quality measures Epstein et al. [47] Cluster RCT, 38 practitioners, 144 patients	ADHD Mean age = 7 years	Collaborative consultation treatment service to promote the use of titration trials and periodic monitoring during medication management vs. control	Audit and feedback Multidisciplinary team	Practitioner adherence/ fidelity as measured by use of titration trials *β* = −0.283; SE, 0.09; *p* < 0.01 and by use of medication monitoring trials: *p* = NS, details NR	Insufficient for practitioner adherence and fidelity	High study limitations, imprecise results (small sample size)
				Lower odds with overlapping confidence intervals of practitioner citing obstacles to implementation of EBP in 6 of 8 measures (2 reached statistical significance)	Insufficient for practitioner competence/ skills	High study limitations, imprecise results (small sample size)
				*F* score for decrease in combined parent and teacher ratings of ADHD symptoms for group x time interaction: *F*_2, 144_ = 0.44, *p* = 0.65	Insufficient for patient change in mental health symptoms	High study limitations, imprecise results (small sample size)
Paying practitioners to implement an EBP Garner et al. [53] Cluster RCT, 105 therapists, 986 patients	Substance use disorders Mean age = 16 years	Paying practitioners for performance in successfully delivering an EBP intervention vs. implementation as usual	Provider incentives	Therapists in the P4P group were over twice as likely to demonstrate implementation competence compared with IAU therapists (Event Rate Ratio, 2.24; 95% CI, 1.12 to 4.48)	Moderate for benefit for practitioner competence	Medium study limitations, precise results
				Patients in the P4P condition were more than 5 times as likely to meet target implementation standards (i.e., to receive specific numbers of treatment procedures and sessions) than IAU patients (OR, 5.19; 95% CI, 1.53 to 17.62)	Low for benefit for practitioner adherence and program fidelity	Medium study limitations, imprecise results (wide CIs)
				No statistically significant differences between groups OR, 0.68; 95% CI, 0.35 to 1.33	Low for no benefit for patient change in mental health symptoms	Medium study limitations, precise results
Program to improve organizational climate and culture Glisson et al. [14]^a^ Two-stage RCT, 596 youth, 257 therapists	Externalizing behaviors (youth referred to juvenile court with behavioral or psychiatric symptoms that require intervention) Ages 9–17 years	Program to improve organizational climate and culture vs. usual care	Educational meetings or materials Educational outreach visits Provider satisfaction initiative Audit and feedback	Details NR but does not demonstrate improvements in any measure of adherence by strategy group for any ARC vs. no ARC comparison	Low for no benefit for practitioner adherence	Medium study limitations, precise results
				Difference in out-of-home placements and child behavior problem scores at 18 months between ARC-only and usual-care groups did not meet statistical significance (*p* = 0.05).	Low for no benefit for patient change in mental health symptoms at 18 months	Medium study limitations, precise results (small sample size), CIs likely overlap
Program to improve organizational climate and culture Glisson et al. [51, 60] Cluster RCT 352 caregivers of youth ages 5–18 in 18 programs	General mental health problems Ages 8–24 years	Program to improve organizational climate and culture vs. usual care	Educational meetings or materials Educational outreach visits Provider satisfaction initiative Audit and feedback	Trends toward improvement in all domains; nonoverlapping CI for some domains showing significant improvements (*p* < 0.05) for ARC group vs. usual care	Low for benefit for practitioner satisfaction	Medium study limitations, imprecise results (small study sample)
				Lower problem behavior scores for youth in the ARC group compared with those in the control group during first 6 months of follow-up (following 18-month organizational implementation), effect size = 0.29	Low for benefit for patient change in mental health symptoms	Medium study limitations, imprecise results (small study sample)
Nurse training to implement an EBP Gully et al. [55] Interrupted time series in Study 1, 172 parents or caregivers; RCT in Study 2, 51 parents or caregivers	General mental health symptoms (children suspected of abuse during forensic medical examinations) Ages 2–17 years	Protocol to train nurses to educate parents about EBPs vs. typical services	Educational meetings or materials Educational outreach visits Patient-reported data	Strategy improved parent ratings of access to care (mean difference between groups ranged from 0.08 to 2.1 points in Study 1 and 0.6 to 1.9 in Study 2) (scale = 1–5)	Low for benefit for patient access to care	High risk of bias, consistent, direct, precise results
				Improved parent ratings of satisfaction of care by a mean of 0.4 in Study 1 and 0.9 in Study 2 (scale = 1–5)	Low for benefit for patient satisfaction	High risk of bias, consistent, direct, precise results
				Improved parent ratings of treatment engagement by a mean of 0.9 in Study 1 and 2.5 in Study 2 (scale = 1–5)	Low for benefit for treatment engagement	High risk of bias, consistent, direct, precise results
				Improved parent ratings of therapeutic alliance by a mean of 0.4 in Study 1 and 0.9 in Study 2 (scale = 1–5)	Low for benefit for therapeutic alliance	High risk of bias, consistent, direct, precise results
Intensive quality assurance to implement an EBP Henggeler et al. [54] Controlled clinical trial, 30 practitioners, N of caregiver and patient reports and monthly data points NR	Substance use disorders (adolescents with marijuana abuse) Ages 12–17 years	Intensive Quality Assurance (IQA) system vs. workshop only to implement an EBP intervention	Quality monitoring	Study does not provide sufficient detail to judge magnitude of effect on practitioner adherence to cognitive behavioral therapy and monitoring techniques	Insufficient for practitioner adherence and fidelity	High study limitations, imprecise results
Training through workshop and resources to implement an EBP Henggeler et al. [59] Cluster RCT; 161 therapists	Substance use disorders Ages 12–17 years	Workshop and resources (WSR) vs. WSR and computer-assisted training (WSR + CAT) to implement an EBP intervention	Educational meetings or materials	No statistically significant difference between groups for use, knowledge, and adherence	Insufficient for additional benefit of WSR + CAT vs. WSR comparison group for practitioner use, knowledge, and adherence	Medium study limitations, imprecise, small sample sizes, cannot determine whether CIs cross line of no difference
		WSR vs. WSR + CAT and supervisory support (WSR + CAT + SS) to implement an EBP intervention	Educational meetings or materials Educational outreach visits	No statistically significant difference between groups for use, knowledge, and adherence	Insufficient for additional benefit of WSR + CAT + SS vs. WSR comparison group on practitioner use, knowledge, and adherence competence/skills	Medium study limitations, imprecise, small sample sizes, cannot determine if CIs cross line of no difference
Professional training to identify and refer cases Lester et al. [48] Cluster RCT; 110 practices, 179 patients	Psychosis (adolescents and adults with first-episode psychosis) Ages 14–30 years	Professional training to identify and refer cases vs. usual care	Educational meetings or materials Educational outreach visits	Relative risk (RR) of referral to early intervention after first contact: 1.20, 95% CI, 0.74 to 1.95, *p* = 0.48	Insufficient for patient access to care	High study limitations, imprecise results
				No statistically significant differences between groups in changes in patient mental health status	Insufficient for patient change in mental health symptoms	High study limitations, imprecise results
				Patients in the professional training group averaged 223.8 fewer days for time from the first decision to seek care to the point of referral to an early intervention service than patients in the control group	Low for benefit for service utilization	High study limitations, imprecise results
				No adverse events were reported, no significant between-group differences for false-positive referral rates from primary care	Insufficient for patient harms	High study limitations, unknown precision
Professional training plus feedback Lochman et al. [57] Cluster RCT, 511 patients	Externalizing behaviors (children at risk for aggressive behaviors) Ages: third-grade students	Professional training plus feedback (CP-TF) to implement an EBP intervention vs. control	Educational meetings or materials Audit and feedback	Students in CP-TF group had fewer behavioral problems as rated by teachers (beta = −0.41, SE = 0.16, *p* = 0.01) than controls but no significant difference in teacher ratings or parent ratings	Low for no benefit for changes in mental health status	Medium study limitations, precise results
				Students in CP-TF group had fewer minor assaults (e.g., hitting or threatening to hit a parent, school staff, or student) as reported by the child (beta = −0.25, SE = 0.12, *p* = 0.03) and social/academic competence as reported by the teacher (beta = 0.35, SE = 0.13, *p* = 0.01) compared with controls	Low for benefit for change in socialization skills and behaviors	Medium study limitations, precise results
		Professional training only to implement an EBP intervention (CF-BT) vs. control	Educational meetings or materials	No significant difference in behavioral problems as rated by teachers or parents or student-reported assaults between CP-BT and control groups	Low for no benefit for changes in mental health status	Medium study limitations, precise results
				No significant differences in social/ academic competence as reported by the teacher, nor were any significant differences found between groups on social skills as rated by parents.	Low for no benefit for change in socialization skills and behaviors	Medium study limitations, precise results
Medication monitoring therapy Ronsley et al., 2012 [49] Interrupted time series Health care practitioners for 2376 patients	Psychosis Ages <19 years (mean age = 11)	Patient medication monitoring training program for practitioners vs. usual care	Educational meetings or materials Educational outreach visits Reminders	38.3% of patients had a metabolic monitoring and documentation tool (MMT) in the charts after program implementation; drop in the prevalence of second-generation antipsychotic prescribing from 15.4% in the pre-metabolic monitoring training program (MMTP) period to 6.4% in the post-MMTP period (*p* < 0.001)	Low for benefit for practitioner adherence	High study limitations, precise outcomes
				Increased metabolic monitoring over time (level of change varied by type of monitoring)	Low for benefit for patient service utilization	High study limitations, precise outcomes
Staffing models to implement an EBP to screen, conduct a brief intervention, and refer adolescents with substance use to treatment from primary care settings Sterling et al. [58] Cluster RCT, 47 pediatricians with 1871 eligible patients	Varied conditions among children attending a pediatric primary care office Ages 12–18	Pediatrician only vs. embedded behavioral health care practitioner (BHCP) implementation of an EBP	Multidisciplinary teams	No significant differences in substance use assessment between study arms (aOR, 0.93; 95% CI, 0.72 to 1.21); patients in the embedded BHCP group more likely than those in the pediatrician-only group to receive brief intervention (aOR = 1.74, 95% CI, 1.31 to 2.31); patients in the BHCP group less likely to receive a referral to a specialist than patients in the primary-care^b^ only group (aOR = 0.58, 95% CI, 0.43 to 0.78)	Low for no benefit for practitioner adherence (2 of 3 adherence outcomes were statistically significant)	Medium study limitations, unable to assess precision
Co-location of a behavioral health EBP parenting program in primary care to help children with externalizing behavioral problems Wildman et al. [52] Controlled clinical trial, 4 pediatric practices, 20,917 children with primary care visit	Externalizing behavior problems Ages 2–12 years	Colocation of a behavioral health EBP parenting program in primary care vs. enhanced referral to a behavioral health EBP parenting program in a location external to the practice.	Changing the scope of benefits	OR for attending first EBP visit, 3.10; 95% CI, 1.63 to 5.89	Low for benefit for patient access to care	High study limitations, precise results
				No improvement in mean number of sessions attended (calculated mean difference: −1.01; 95% CI, −2.60 to 0.58)	Insufficient for patient service utilization	High study limitations, precise results
Implementation of a school-based cognitive-behavioral group EBP Warner et al. [62] Stratified RCT 138 youth, 7 master’s level school counselors, 5 doctoral-level psychologists	Social anxiety disorder. Adolescents in grades 9–11 from three suburban public high schools identified via school-wide screening, parent telephone screening, and clinical diagnostic evaluation with no other mental disorders of equal or greater severity.	Implementation by a school counselor vs. by a psychologist	Changing provider	No significant differences in implementation adherence or competence.	Insufficient for practitioner adherence or competence	High study limitations, unknown precision for each intermediate outcome
				No significant differences between groups for any of the severity or functioning scales at post-treatment or follow-up with the exception of 3 posttreatment outcomes (treatment response, treatment remission and social anxiety severity as rated by parents) where youth in the school counselors group did not do as well as those in the psychologist group when noninferiority was tested	Insufficient for patient change in mental health status	High study limitations, unknown precision for each intermediate outcome

^a^Four study groups were examined: ARC + MST, ARC only, MST only, and usual care. Comparisons were ARC only vs. usual care or any ARC (combined ARC + MST and ARC only) vs. no ARC (combined MST and usual care), as noted

^b^Fewer referrals seen as improvement because this outcome indicates that the practitioner was able to give brief intervention without referral to behavioral health specialists

*ADHD* attention deficit hyperactivity disorder, *aOR* adjusted odds ratio, *ARC* Availability, Responsiveness, and Continuity, *CBT* cognitive behavioral therapy, *CI* confidence interval, *CP-TF* Coping Power training plus feedback, *EBP* evidence-based practice, *EHR* electronic health record, *IAU* implementation as usual, *IQA* Intensive Quality Assurance, *MMT* metabolic monitoring program, *MMTP* metabolic monitoring training program, *MST* multisystemic therapy, *N* number, *NR* not reported, *NS* not significant, *OR* odds ratio, *p* probability, *P4P* pay for performance, *RCT* randomized controlled trial, *RR* relative risk, *SE* standard error, *WSR* workshop plus resources, *WSR + CAT* workshop plus resources plus computer-assisted training, *WSR + CAT + SS* workshop plus resources plus computer-assisted training plus supervisory support

The strongest evidence in the review (for KQ 1) comes from a study of pay for performance. Therapists in the pay for performance group were more than twice as likely to demonstrate implementation competence (as were therapists in the implementation as usual group (moderate strength of evidence of benefit) [53]. In this instance, implementation competence was defined as therapists demonstrating competent delivery of all components of at least one Adolescent Community Reinforcement Approach (A-CRA, an EBP program) treatment procedure during the same treatment session for 1 month. Other outcomes for which we found evidence of benefit (low strength of evidence of benefit) included:

1. Improved practitioner adherence to EBPs or guidelines: by training practitioners to monitor metabolic markers [49], providing computer decision support plus an electronic health record (EHR) that included diagnosis and treatment guidelines [46], or offering an Internet portal for practitioner access to practice guidelines [56]
2. Improved practitioner morale, engagement, and stress: by implementing a program to enhance organizational climate and culture [51]
3. Improved patient access to care, parent satisfaction, treatment engagement, and therapeutic alliance: by training nurses to educate parents about EBPs [55]
4. Improved patient functional status: by giving practitioners weekly feedback on patient symptoms and functioning [13]
5. Improved service utilization: by training practitioners about monitoring medications [49] and appropriately identifying and referring patients with mental health problems [48].

Six strategies (one study each) consistently provided insufficient evidence or evidence of no benefit across all reported outcomes. These included:

1. A strategy to test augmented active learning versus computerized routine learning versus routine practitioner workshop to implement an EBP [50]
2. A collaborative consultation treatment service to promote the use of titration trials and periodic monitoring during medication management versus control [47]
3. An Intensive Quality Assurance system versus workshop to implement an EBP intervention [54]
4. Use of additional computerized assisted training or computerized training plus supervisory support to implement an EBP versus using a workshop and resources only [59]
5. A Contextualized Feedback Systems trial to test the provision of session-by-session clinician feedback versus clinician feedback provided after 6 months of visits
6. A strategy to test the implementation of a school-based EBP by school counselors versus psychologists

The absence of evidence on several factors of interest further limited our conclusions. We found no evidence of studies examining several intermediate outcomes, particularly system-level intermediate outcomes. In addition, we identified no studies that measured final patient health outcomes such as co-occurring conditions. We also found no evidence of strategies testing several components of the EPOC taxonomy, including any regulatory components, and little evidence on strategies with financial components.

Of the 19 studies in our review, we rated risk of bias as follows: low, one study; medium, five studies; high, six studies; and unclear, seven studies. Various issues with study design, attrition, and incomplete information reported by study authors explain the risk of bias issues for high and unclear ratings.

The uncertain or high risk of bias of most of these studies affected the overall strength of evidence grades, as did the fact that generally we had only single studies for each strategy examined.

Only one study evaluated any harms (KQ 2), in this case associated with professional training to identify and refer cases to early-intervention services for untreated first-episode cases of psychosis [48]. The study reported no adverse events and no differences between strategy and control groups in false-positive referral rates. We graded the evidence on harms as insufficient, based on high study limitations and imprecise results.

Overall, we found evidence on four strategies that examined moderators of the effectiveness of strategies to improve mental health care for children and adolescents (KQ 3). Three examined whether training intensity influenced the degree of effectiveness; of these, two strategies were graded as having insufficient strength of evidence. The third strategy had low strength of evidence for benefit for patient intermediate outcomes (access to care) and patient health outcomes (change in mental health status).

A fourth study examined the moderating effects of fidelity to the EBP (A-CRA) in therapists meeting individual patient targets specified in the A-CRA program. We graded this evidence about fidelity as low strength for no benefit on patient health outcomes and patient remission status.

We did not find studies that examined most of our previously specified list of moderators—namely, as patient characteristics, intervention characteristics other than training intensity, outer context, inner context, characteristics of involved individuals, process characteristics other than fidelity to the training, or other moderators such as length of follow-up.

### Additional qualitative synthesis

**Fig. 3:**
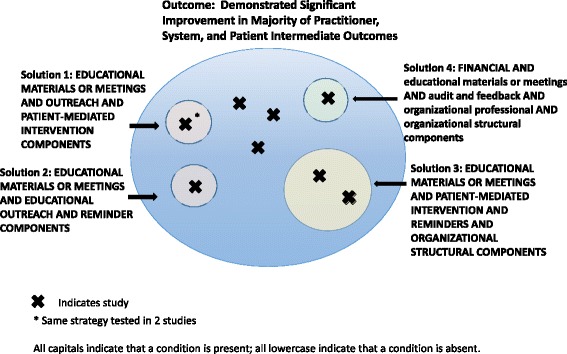
Qualitative comparative analysis findings

To understand better what combinations of components (“condition sets”) might serve as solutions or “recipes for success”, we turned to QCA. We examined several different models that had different combinations of intervention components; we tested two different outcomes. We chose the model that best fit our data with the highest level of consistency (proportion of solutions resulting in success or outcome) and coverage (proportion of observations explained by the solutions). Our model included the presence or absence of several professional components (educational materials or meetings, educational outreach, patient-mediated interventions, audit and feedback), any financial component, organizational structural-oriented components (quality monitoring or change in scope and nature of benefits and services), and organizational provider-oriented component (use of clinical multidisciplinary teams, provider satisfaction with conditions of work and the material or psychic rewards, or revision of professional roles). We defined success as having a statistically significant improvement in either a majority of practitioner-, system-, and patient-level intermediate outcomes or at least one patient health or service utilization outcome with at least low strength of evidence for benefit (Fig. 3).

Our analysis included 19 studies; 9 of 18 studies showed at least low strength of evidence of benefit and significant improvements in the majority of practitioner, system, or patient intermediate outcomes tested (one study did not test any intermediate outcomes). An additional 3 of the 10 remaining studies showed at least low strength of evidence of benefit for at least one patient health or service utilization outcome. Seven studies did not meet either of these benefit criteria.

In additional analyses, no conditions were individually necessary or sufficient, and no necessary combinations occurred. Analysis of sufficient combinations for achieving significant improvements showed four solutions, each with 100% consistency. Notably, the model had 67% coverage, accounting for 6 of the 9 studies that demonstrated at least low strength of evidence of benefit for the majority of intermediate outcomes tested. These complex solutions were:

1. Having educational materials or meetings, educational outreach, and patient-mediated intervention components
2. Having educational materials or meetings, educational outreach, and reminder components
3. Having educational materials or meetings, patient-mediated intervention components, reminders, and organizational structural components (quality monitoring or change in scope and nature of benefits and services)
4. Having a financial component and *not* educational materials or meetings, *not* audit and feedback, *not* organizational structural components, and *not* organizational provider components (multidisciplinary teams, provider satisfaction, or revision of professional roles

Because only one strategy, pay for performance, contained a financial component and no other components, we reran the without including this study in the analyses as a sensitivity analysis. The first three solutions above remained, which covered five of the eight studies that demonstrated at least low strength of evidence of benefit for the majority of intermediate outcomes tested.

## Discussion

Overall, 12 of the 19 studies demonstrated effectiveness as measured by moderate or mainly low strength of evidence for benefit for at least one outcome of interest. Our confidence in these results is limited by the paucity of studies on any strategy. We graded benefits of pay for performance as moderate strength of evidence [53]. We graded the strength of evidence of benefit as low for at least one outcome among strategies that contained:

- Reminders (i.e., a component that included patient- or encounter-specific information, provided verbally, on paper, or on a computer screen, that was designed or intended to prompt a health professional to recall information) [46, 49, 56]
- A patient-mediated component (i.e., one that collected new clinical information directly from patients that was then given to the provider to review) [46, 55, 56]
- Enhanced referrals and patient choice of treatment [52]

We found low strength of evidence of no benefit for intermediate outcomes for strategies that involved the following combinations of professional components:

- Educational materials and/or educational meeting components only (i.e., no other components) [50, 59]
- Educational materials and outreach components only (i.e., no other components) [48, 57]

We were unable to judge the potential for harms associated with these strategies that might mitigate benefits. We had only a single study on early intervention for first-episode psychosis, and it reported no adverse events and no differences in false-positive referral rates. In addition, the available evidence from four studies on two moderators does not permit us to make general conclusions about the conditions under which these strategies might work optimally.

The studies varied with respect to the numbers and types of active components, i.e., we observed considerable differences in components in treatment group strategies and comparison group strategies. In some studies, the treatment group contained several components and the comparison group contained none of those components. In other studies, both the treatment and comparison groups tested strategies with multiple components, with different numbers of variations in components across arms. Because both arms often received active interventions, the Hawthorne effect may explain lack of effectiveness. We did not find any consistent patterns of effectiveness involving the number of active components. That is, we did not find that studies that employed strategies with a single active component had any better or any worse effect on outcomes than those that employed multiple active components.

Additional heterogeneity arose from several other sources and precluded any quantitative synthesis of our findings. Except for two studies reported in one publication [55], two trials (three publications) reporting variants of a similar strategy [14, 51, 60], and two trials reporting different types of feedback strategies [13, 61], no other studies tested similar strategies. The outcomes of the studies varied widely. Similarly, settings differed greatly (community-based hospitals and clinics, general practice and primary care, home-based mental health systems, schools, substance abuse treatment). Finally, the targets of each strategy, such as practitioners, practices, or systems, also differed considerably.

Challenges in this systematic review arose with defining the intervention of interest, constructing the search strategy, and applying prespecified inclusion/exclusion criteria. The lack of consistency in the terminology used in the published literature meant that the use of self-selected descriptors such as “QI,” “implementation,” or “dissemination” by study authors did not conform either to our a priori definitions of these types of studies or to the other similarly labeled studies in the field. This lack of consistency led to our reliance on the EPOC taxonomy as our primary analytic framework.

Strategies differed considerably in the number of components; the reporting on these components was not always clear enough either to describe the strategy adequately or to let us understand fully the relative importance of component parts. New taxonomies are continually emerging, such as the revised EPOC taxonomy [63] and the refined compilation of implementation strategies from the Expert Recommendations for Implementing Change (ERIC) project [64]. Both might help advance the field by clarifying the conceptual models that underlie this research.

Trying to specify the population and comparison criteria to ensure greater homogeneity of included interventions posed additional challenges. For example, our focus on children and adolescents with *existing* mental health issues (rather than only the *risk of* mental health issues) meant we could not examine issues of prevention. In addition, although we included a broad range of eligible comparators in our protocol (usual care, or any other QI, implementation, or dissemination strategy), we did encounter otherwise eligible studies in which the intervention combined both a patient-level intervention and a system-level strategy to implement or disseminate that intervention. Because the use of a “usual-care” arm did not permit the original investigators to draw conclusions about the effect of their implementation or dissemination strategy apart from the underlying intervention, we excluded these studies for having a wrong comparator [65–72]. In addition, studies often offered limited descriptions of usual-care arms relative to descriptions of experimental arms. Even with incomplete reporting, however, we found wide differences in the number, intensity, and services offered in usual-care arms. These differences sharply restricted our ability to make statements about the overall effectiveness of these strategies as a class.

Reporting issues in the literature also hindered our ability to derive firm conclusions on the effectiveness of included strategies. Authors reported complex analyses but often did not report other aspects of their methods well enough to permit an independent evaluation of the effect size [57], precision of the effect [46–48, 51], or risk of bias [46, 57].

We did not find evidence on the majority of the outcomes that we had specified a priori. Of particular note, seven strategies (two from a single publication) relied on EBPs and did not report patient health outcomes [50, 54–56, 58, 59].

When researchers maintain fidelity to the original intervention, assuming that the same level of effectiveness will occur in a new trial is reasonable; adopting this assumption can, therefore, produce efficient use of research funds. Unfortunately, not all studies measured fidelity adequately. New strategies relying on EBPs must, at a minimum, report on fidelity so that practitioners and policymakers can judge whether the strategy is, in fact, a new intervention rather than implementation or dissemination of an existing intervention. Information on pragmatic issues related to implementation (fidelity, adaptation, and minimum elements necessary to achieve change) may not necessarily require new studies on strategies with existing information; support of analyses done with data from existing studies may fill some of the gap.

The sparse evidence base of a set of diverse strategies and outcomes focusing on intermediate and health outcomes and resource use highlights the fact that clinicians and health plan administrators still do not have adequate knowledge of best methods to introduce EBPs successfully into clinical settings for children and adolescent populations. Third-party payers are paying increasing attention to quality metrics, as health care systems move to accountable care models. We found no studies on regulatory components and just one study testing the effectiveness of a financial component, specifically for pay for performance [56]. Future research efforts should evaluate variations of such programs according to patient, provider, organization, systems, and setting characteristics. A better understanding of these variables may help promote the implementation and dissemination of EBPs.

The majority of included studies appropriately used cluster designs. Cluster RCTs, like pragmatic trials, typically need more resources than conventional RCTs. Moreover, they can be harder to analyze than conventional studies. An additional consideration (for all these trials) related to inadequate reporting, a problem often noted in the literature [73, 74]. The studies we found were marked by poor reporting or failure to report key details of the strategy or differences across study arms. A recently developed tool, the StaRI (standards for reporting implementation studies of complex interventions), offers standards for reporting implementation studies [75]. If adopted widely, StaRI may well considerably improve the utility of these studies and the pace of translation of evidence into practice.

Although the failure to use EBPs can lead to gaps between potential and achieved outcomes, closing such holes in the knowledge base requires more than just using an array of EBPs. What continues to be unknown is how to bridge the gap in the context of the finite resource of time allocated for a patient encounter and what implications changes will have on current work flow processes. As expectations increase for documenting or checking off quality metrics for each action within a patient encounter, the risks of errors of both omission and commission rise. For new information to be actionable, more persuasive evidence is needed on the relative merits of each action or strategy.

More research is needed on strategies for the QI, implementation, and dissemination of EBPs relating to both psychological and behavioral treatments and medication interventions for treating youth suffering from mental illness. Other important research targets include developing and testing dissemination strategies for introducing mental health care into geographic areas lacking in mental health care, such as very rural areas with fewer mental health providers than more urban locations have. In these areas especially, focusing more attention on primary care providers may be essential.

## Conclusions

The evidence does not give us a high degree of confidence about the efficacy of any one strategy to improve the implementation, dissemination, or quality of mental health care for children and adolescents, generally because we had only a single study testing a given strategy. Although we had insufficient or low strength of evidence of no benefit for more than half of the outcomes that we evaluated, our findings suggest that several approaches can improve both intermediate and final health outcomes and resource use. Of the 19 included studies, 12 significantly improved at least one such outcome or measure. Moderate strength of evidence (from one RCT) supported using provider financial incentives such as pay for performance to improve the competence with which practitioners can implement EBPs. We found inconsistent evidence involving strategies with educational meetings or materials; programs appeared to be successful in combination with outreach, reminders, providing practitioners with newly collected clinical information, or organizational structural components including quality monitoring and changing the scope or nature of benefits. We also found low strength of evidence for *no* benefit for initiatives that included only educational materials or meetings (or both) or only organizational provider components.

## Abbreviations

ACROBAT-NRSI: A Cochrane Risk Of Bias Assessment Tool for Non-Randomized Studies of Interventions
ADHD: Attention deficit hyperactivity disorder
AHRQ: Agency for Healthcare Research and Quality
aOR: Adjusted odds ratio
ARC: Availability, Responsiveness and Continuity
CBT: Cognitive behavioral therapy
CCT: Controlled clinical trials (nonrandomized)
CFIR: Consolidated Framework for Implementation Research
CI: Confidence interval
CINAHL: Cumulative Index to Nursing and Allied Health Literature
CP-TF: Coping Power training plus feedback
D: Dissemination
EBP: Evidence-based practice
EHR: Electronic Health Record
EPC: Evidence-based Practice Center
EPOC: Effective Practice and Organization of Care
HDI: Human Development Index
I: Implementation
IAU: Implementation as usual
IQA: Intensive Quality Assurance
KQ: Key question
MeSH®: Medical Subject Headings
MMT: Metabolic monitoring program
MMTP: Metabolic monitoring training program
MST: Multisystemic therapy
N: Number
NR: Not reported
NREPP: National Registry of Evidence-based Programs and Practices
NS: Not significant
OR: Odds ratio
P: Probability
P4P: Pay for performance
PICOTS: Patient population, intervention, comparator, outcome, timing of outcome assessment, and setting
PRISMA: Preferred Reporting Items for Systematic Reviews and Meta-Analyses
QCA: Qualitative comparative analysis
QI: Quality improvement
RCT: Randomized controlled trials
RR: Relative risk
SAMHSA: Substance Abuse and Mental Health Services Administration
SE: Standard error
StaRI: Standards for reporting implementation studies of complex interventions
WSR + CAT + SS: Workshop plus resources plus computer-assisted training plus supervisory support
WSR + CAT: Workshop plus resources plus computer-assisted training
WSR: Workshop plus resources
